# Supplementary material for: Genome-Wide Identification and Analysis of the BBX Gene Family and Its Role in Carotenoid Biosynthesis in Wolfberry (Lycium barbarum L.)
Source: Int J Mol Sci. 2022 Jul 29;23(15):8440. doi: 10.3390/ijms23158440 (PMC9369241; doi:10.3390/ijms23158440)
Supplement: Supplementary file 1 [file ijms-23-08440-s001.zip › Supplementary Figures S1--S4.pdf]

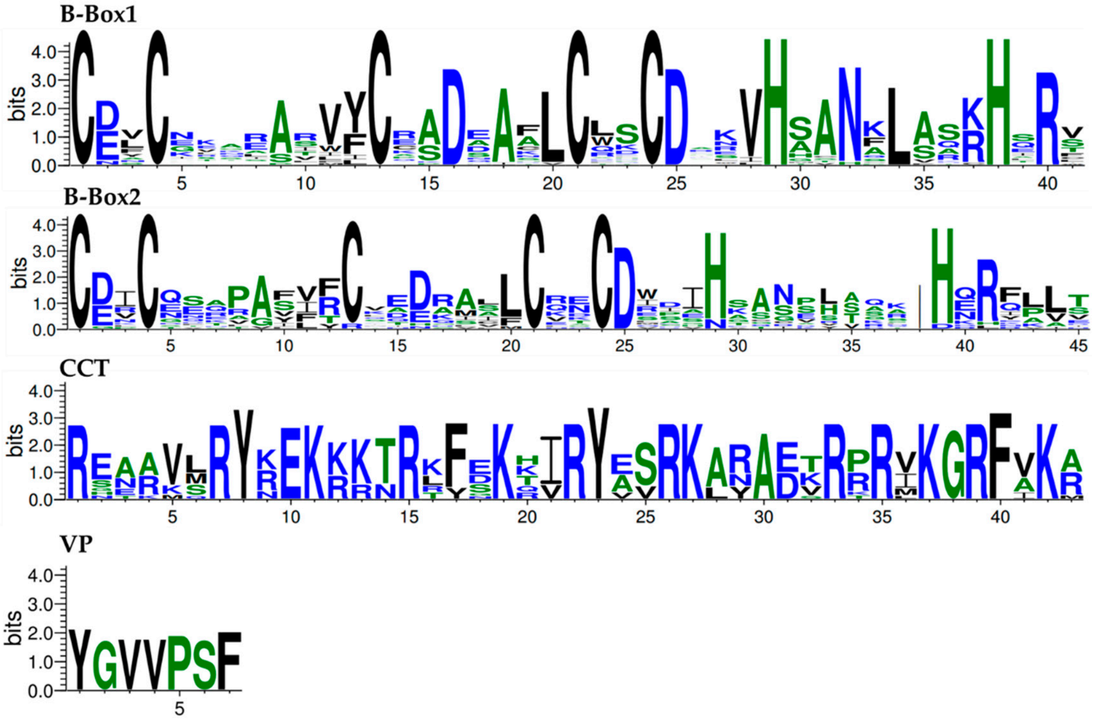

Figure S1. Domain compositions of LbaBBX proteins. The amino acid sequence alignment of the B-box1, B-box2, CCT, and VP domain. The x-axis indicated the conserved sequences of the domain and the conservation rate of each amino acid, respectively.

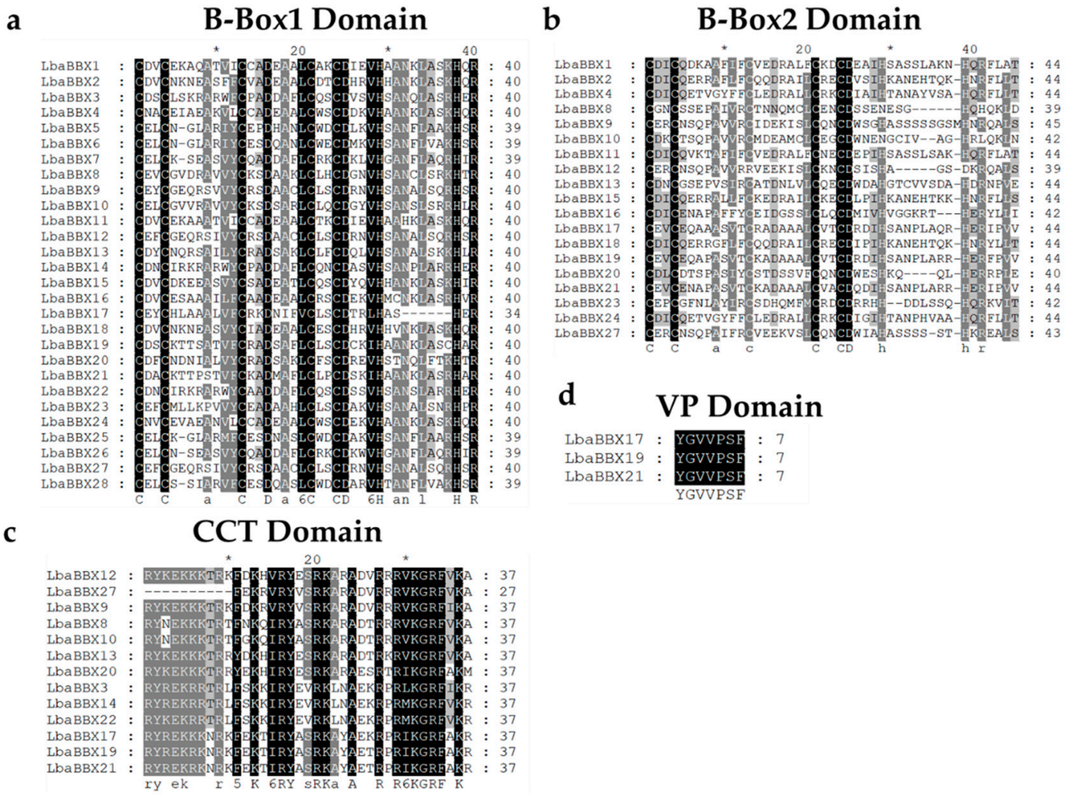

Figure S2. Multiple sequence alignments of the domains of the LbaBBX proteins. (a), (b), (c), and (d) represent the protein alignment of the B-Box 1, B-Box 2, CCT and VP domain, respectively. The identical conserved amino acids were represented by black and gray shaded.

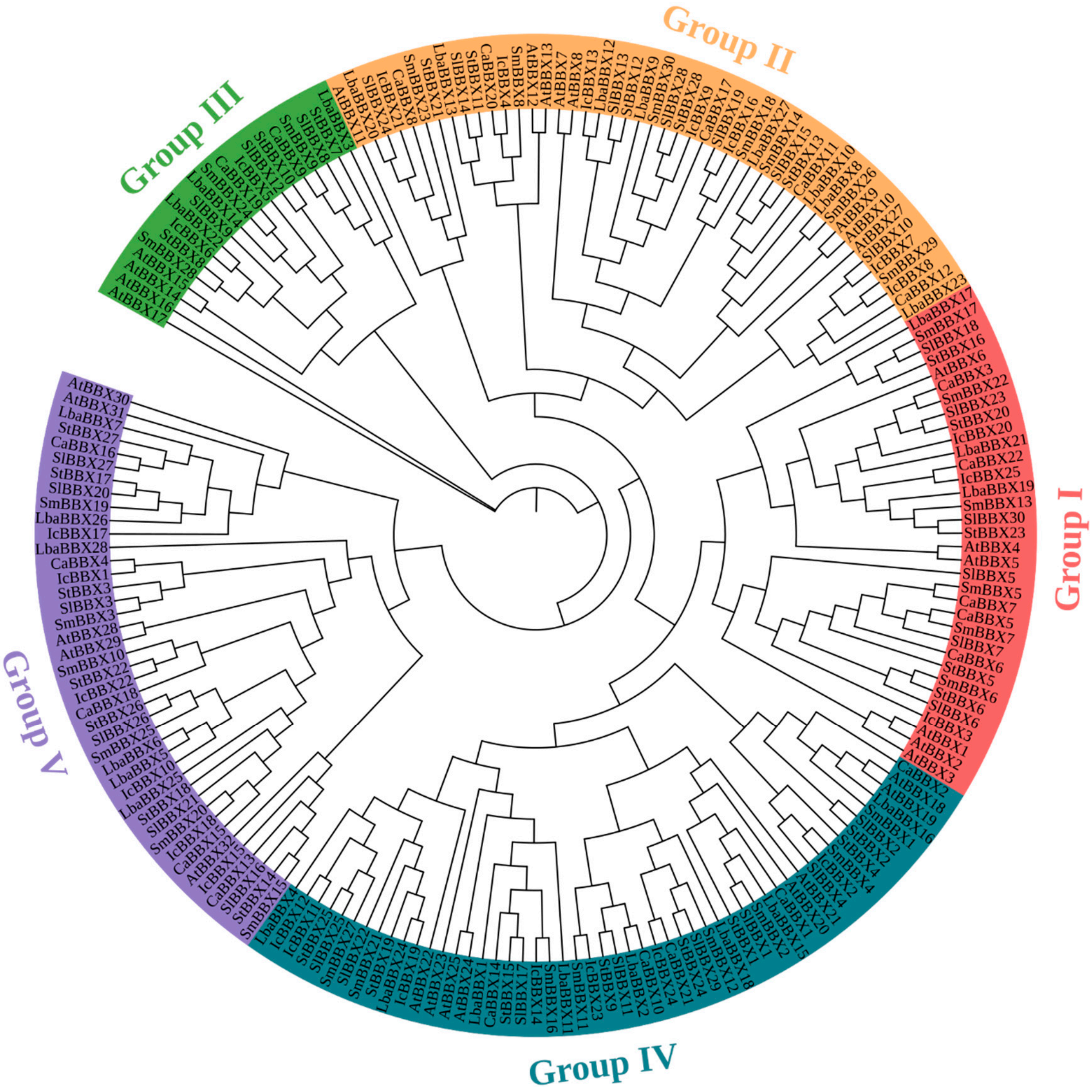

Figure S3. Phylogenetic analysis tree of BBX proteins from Arabidopsis and six Solanaceae species, including wolfberry (*Lycium barbarum*), tomato (*Solanum lycopersicum*), pepper (*Capsicum annuum*), eggplant (*Solanum melongena*), potato (*Solanum tuberosum*), and *Iochroma cyaneum.* (At represent *Arabidopsis thaliana*; Lba represent *Lycium barbarum*; Sl represent *Solanum lycopersicum*; Ca represent *Capsicum annuum*; Sm represent *Solanum melongena; St* represent *Solanum tuberosum*; IC represent *Iochroma cyaneum*, respectively. The full-length amino acid sequences of BBX protein were aligned by Muscle, and the phylogenetic tree was generated using the Maximum likelihood (ML) method with 1000 bootstrap replicates by IQ-TREE. The tree divided into five groups, which are marked by different colors and named as Group I, II, III, IV, and V.

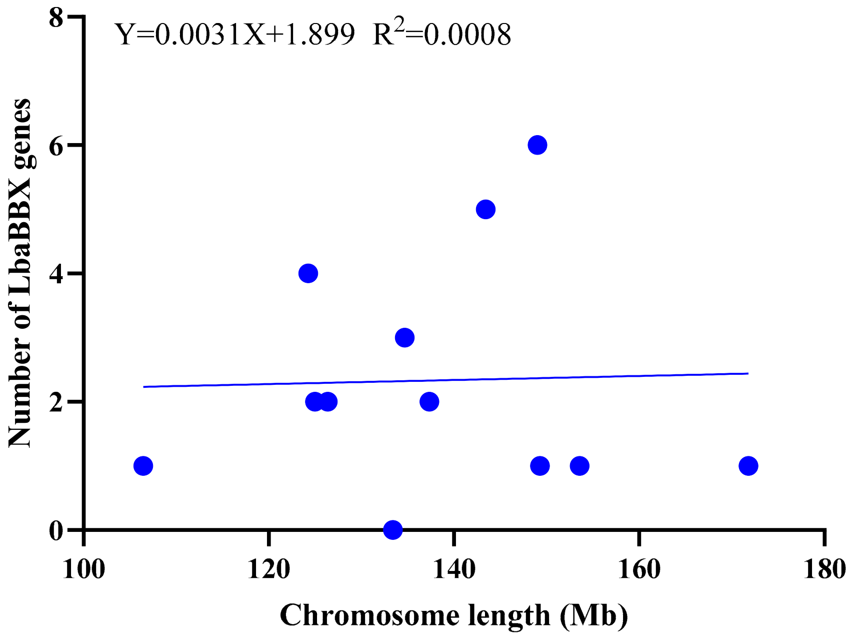

Figure S4. The Relationship between the numbers of *LbaBBX* genes and chromosome length.
